# Comparison of muscle oxygenation and total hemoglobin levels during isokinetic concentric and eccentric contractions at different speeds in athletes

**DOI:** 10.1186/s13102-026-01630-y

**Published:** 2026-03-02

**Authors:** Dur Samand, Nevin A. Guzel, Gamze Cobanoglu

**Affiliations:** 1 Department of Physiotherapy and Rehabilitation, Institute of Health Sciences, Gazi University, Ankara, Turkey; 2 Department of Physiotherapy and Rehabilitation, Faculty of Health Sciences, Gazi University, Ankara, Turkey

**Keywords:** Near infrared spectroscopy, Muscle oxygen saturation, Total hemoglobin level. Isokinetic, Volleyball athletes

## Abstract

**Objective:**

This cross-sectional observational study examined muscle oxygen saturation (SmO₂) and additional Near-Infrared Spectroscopy (NIRS)-derived parameters—oxygenated hemoglobin (O₂Hb), deoxygenated hemoglobin (HHb), hemoglobin difference (Hb Diff), and total hemoglobin levels (tHb)—in the vastus lateralis (VL) and biceps femoris (BF) muscles between athletes and sedentary individuals during isokinetic maximal concentric (CON) and eccentric (ECC) contractions executed at 60, 180, and 300°/s speeds.

**Methods:**

Thirty-one male participants (17 elite volleyball athletes, 14 sedentary) underwent a testing protocol using an isokinetic dynamometer. NIRS sensors recorded SmO₂, O₂Hb, HHb, Hb Diff, and tHb on the VL and BF during maximal CON and ECC sets at each speed. Two-way repeated-measures ANOVA (speed × group × contraction) was applied; normality was verified (Shapiro–Wilk), and Greenhouse–Geisser corrections were used if needed. Data are reported as mean ± SD.

**Results:**

In the VL, high speed (300°/s) significantly raised SmO₂ and tHb relative to 60°/s (*p* < 0.05). In BF, SmO₂ was stable across speeds, although Hb Diff increased at 300°/s (*p* = 0.008). At 60°/s, VL CON produced greater tHb, O₂Hb, and Hb Diff than ECC (*p* < 0.001); in BF at 60°/s, ECC produced higher O₂Hb and Hb Diff than CON (*p* < 0.001). Athletes had higher VL tHb and O₂Hb than sedentary individuals (*p* < 0.05); BF group differences were minimal.

**Conclusions:**

Isokinetic velocity and contraction mode significantly altered muscle oxygenation in a muscle-specific manner. High-speed isokinetic CON exercises may enhance oxygenation and local blood flow in the VL of the quadriceps, potentially supporting muscle function, while the hamstrings’ limited responsiveness to speed variations highlights the need for alternative strategies. CON vs. ECC actions also elicited distinct oxygenation in each muscle, reflecting differences in contraction-specific physiological demands. These findings – based on measured NIRS variables – highlight how velocity, contraction type, and training status modulate muscle oxygen dynamics, providing a physiological basis for tailoring training and rehabilitation protocols.

## Introduction

Isokinetic dynamometers are specialized systems that enable the controlled production of muscle contraction patterns at varied speeds, allowing for the real-time assessment of muscle strength, power, and endurance [1]. These devices permit the evaluation of concentric (CON) and eccentric (ECC) contractions under standardized mechanical conditions. CON and ECC contractions differ significantly in their demands on muscle blood supply. While ECC contractions typically use less oxygen, they may cause greater muscle stress, whereas CON contractions demand a higher blood flow to sustain energy turnover [2].

To assess the associated muscle oxygenation dynamics during these controlled contractions performed by isokinetic system, near-infrared spectroscopy (NIRS) can be used [3]. NIRS has gained popularity due to its ability to assess local muscle oxidative metabolism across various exercise modalities [3]. As a non-invasive optical technique that uses infrared light to pass through biological tissues, NIRS allows the monitoring of oxygenated hemoglobin saturation in the tissue, often referred to as muscle oxygen saturation (SmO₂) [4]. SmO₂ reflects the balance between oxygen delivery and consumption within the muscle [5] providing valuable information for optimizing training loads and rehabilitation protocols [6]. However, SmO₂ is a composite signal influenced by oxygen delivery, extraction, and local blood volume. Although perfusion can often be inferred indirectly from SmO₂, this inference becomes less reliable during maximal isokinetic contractions, where contraction mode and angular velocity can mechanically alter intramuscular pressure and microvascular blood volume partly independently of metabolic demand [7]. Therefore, similar SmO₂ responses may arise from different underlying physiological mechanisms.

For this reason, the concurrent assessment of additional NIRS-derived parameters—oxygenated hemoglobin (O₂Hb), deoxygenated hemoglobin (HHb), hemoglobin difference (Hb Diff), and total hemoglobin (tHb)—is warranted to better contextualize SmO₂ changes [3]. O₂Hb represents oxygenated hemoglobin, indicating the amount of oxygen delivered to the muscle, while HHb reflects oxygen consumption by the muscle tissue [8]. HHb is commonly interpreted as a marker of oxygen extraction; however, during dynamic exercise, its sensitivity may be limited by changes in blood volume and NIRS signal characteristics. Hb Diff provides insight into net oxygen extraction and is particularly sensitive to changes in metabolic demand when blood volume remains relatively stable [9]. The tHb, calculated from the sum of O₂Hb and HHb [4], indicates alterations in local blood volume [5]. Literature has reported a strong relationship between the NIRS-derived tHb measure and blood flow measurements obtained via Doppler ultrasound, particularly during dynamic resistance exercise [5], suggesting that tHb responses reliably reflect changes in blood flow within microcirculation.

Muscle composition and fitness level further influence oxygenation, with athletes typically exhibiting greater oxygenation capacity than sedentary individuals, likely due to structural and functional skeletal muscle adaptations [10]. Volleyball athletes represent a population exposed to repetitive high-intensity lower-limb loading, rapid force production, and frequent eccentric actions during jumping and landing tasks. These sport-specific demands may induce distinct neuromuscular and vascular adaptations in muscles. Comparing volleyball athletes with sedentary individuals therefore provides a relevant model for examining how long-term training adaptations influence muscle oxygenation and blood volume responses under controlled isokinetic conditions.

Despite the growing use of NIRS, there remains a lack of comprehensive data examining the combined SmO₂, O₂Hb, HHb, Hb Diff, and tHb during isokinetic CON and ECC contractions across different angular velocities. Clarifying how contraction velocity, contraction mode, and training status influence muscle-specific oxygenation and blood volume responses is essential for optimizing exercise prescription and rehabilitation strategies. Understanding these physiological differences can inform the selection of contraction types and movement speeds that appropriately balance mechanical loading and metabolic stress, thereby enhancing performance adaptations and reducing injury risk.

Therefore, the purpose of this research was to explore SmO₂ and tHb dynamics along with NIRS-derived parameters in the vastus lateralis (VL) and biceps femoris (BF) muscles during maximal CON and ECC contractions at speeds of 60, 180, and 300°/s between athletes and sedentary individuals. We hypothesized that these physiological responses would vary based on contraction type, speed, and training background, providing insights into optimizing exercise and rehabilitation protocols.

## Methods

### Participants

This observational, cross-sectional study was conducted within the Athlete Health Unit of the Department of Physiotherapy and Rehabilitation at the University’s Faculty of Health Sciences and is reported in accordance with the Strengthening the Reporting of Observational Studies in Epidemiology (STROBE) guidelines. Ethical approval for the study (Approval No: 2023 − 1142) was obtained from the Gazi University Ethics Commission, Gazi University, on October 5, 2023, and informed consent was obtained from all participants prior to their inclusion.

This study involved 17 licensed elite volleyball athletes and 14 healthy sedentary individuals. The participants met the following criteria: elite athletes aged 18–35, with a body mass index (BMI) of under 30 kg/m², body fat percentage below 35%, and adipose tissue thickness of ≤ 17.5 mm. Exclusion criteria for all participants included any lower limb injuries or pain in the past 3 months, chronic diseases, smoking, and the use of performance-enhancing substances. The sedentary group met the same criteria, except they were classified as inactive according to the International Physical Activity Questionnaire (IPAQ) Turkish short form [11]. The questionnaire contained seven questions. Participants reported the frequency and duration of their physical activities across different intensity levels [11]. Subjects were then categorized into three categories: Inactive, Minimally Active, and Highly Active, based on their weekly total metabolic equivalent (MET-min/week) as per the original IPAQ scoring protocol [12]. Participants with little to no physical activity were categorized into the sedentary group.

### Protocols

Before the assessment, the individual’s details, including their full name, age, height, profession, years of involvement in sports, specific positions played, and any past injuries, were gathered verbally and documented on the evaluation form. They underwent a 5-minute warm-up session using a bicycle ergometer before the evaluations. Then, participants underwent segmental body analysis using the body composition analyzer. Finally, they were positioned on the isokinetic dynamometer to perform the test protocol, adhering to the manufacturer’s guidelines for proper alignment and stability. Before placing the NIRS device, skinfold thickness was measured over the VL and BF muscles was measured using a caliper.

### Analysis of body composition

Before taking measurements, participants were given clear instructions to reduce any potential fluctuations in impedance readings caused by shifts in body fluid distribution. Body composition was assessed using an analyzer (TANITA BC-418, Tanita Corporation, Japan), following the protocol described by previous researchers [13]. Only data related to total body weight, percentage of body fat, and BMI were included in the analysis.

### Measurement of skinfold thickness

Skinfold thickness over the VL and BF muscles was measured using a caliper (Harpenden Skinfold Caliper 12–1110, Baty International, UK). Due to its potential impact on NIRS signal amplitude, we needed to calculate the thickness of adipose tissue, which is half of the skinfold depth [14]. Hence, the measured skinfold thickness was divided by two.

For the BF and VL muscles, the skinfold thickness was determined by averaging two measurements [15]. The measurements were conducted at the NIRS device location using a caliper. These measurements adhered to the guidelines in The International Society for the Advancement of Kinanthropometry (ISAK) protocol [16, 17]. These measurements were used as control variables to ensure the validity of the NIRS signal, rather than as outcome measures.

### Isokinetic testing

Two different types of contractions, such as CON and ECC, were conducted on the quadriceps and hamstring muscles. This was done using an isokinetic dynamometer (CSMI Cybex Humac Norm, USA). The evaluation comprised two parts: assessing the quadriceps muscles in a seated position and evaluating the hamstring muscles in a prone position. The dynamometer calibration was adjusted as per the manufacturer’s guidelines.

The quadriceps muscle was assessed through a series of tests conducted at varying speeds. Five repetitions were performed at a velocity of 60 degrees per second (°/s) (low-speed), 10 repetitions at 180°/s (medium-speed), and 15 repetitions at 300°/s (high-speed) in maximal CON mode [18]. Afterward, five repetitions at a speed of 60°/s were performed for maximal ECC contraction. For the hamstring muscle, in the prone position, the pelvis was secured with a belt, aligning the hip at 180° and the same testing protocol as the quadriceps was followed [19]. Different repetition numbers were selected across varying speeds following established isokinetic testing protocols used to assess knee muscle performance.

The extent of knee movement, i.e., range of motion, was established. It was set from a flexed position of 90 degrees to an extended position of 0 degrees for CON contractions and from 90° to 10° for ECC contractions [20]. Prior to commencing the evaluation at each velocity, participants performed three practice repetitions at submaximal intensity (equivalent to 50% of their self-perceived effort) to ensure task familiarization [21]. A 30-second break was given during the intervals between submaximal attempts. Additionally, a 1-minute rest period was provided between each testing speed, and a 10-minute rest period was given between quadriceps and hamstring assessments to reduce potential fatigue effects [22]. The testing order was randomized across conditions to minimize order effects and standardized verbal instructions were provided to participants [21]. Peak torque at each repetition for each isokinetic angular velocity was recorded in Nm/kg [20].

### NIRS measurement

A NIRS device (Train.Red FYER, Netherlands) was used in this study. According to the manufacturer, the device uses a light-emitting diode operating at 760 and 850 nm and a line receiver with a reported midrange pixel distance of 35 mm [23]. However, direct equivalence between devices cannot be assumed, and device-specific validation of the Train.Red FYER itself, particularly in dynamic lower-limb applications, remains necessary.

In general, NIRS theory describes measurement sensitivity as arising from a probabilistic tissue volume influenced by optical properties, wavelength, and source–detector geometry, rather than from a fixed penetration depth [24, 25]. However, such theoretical principles cannot be assumed to apply uniformly across different NIRS systems, as device-specific hardware design and signal processing algorithms may differ. Therefore, NIRS-derived measures should be interpreted within the context of the specific device used in this study.

To minimize superficial tissue contribution, all participants had subcutaneous fat thickness below 17.5 mm at the location of the NIRS device. The NIRS sensor was placed on the participant’s dominant leg during the testing session, specifically targeting the VL and BF, utilizing a specially designed adaptable band. The positioning for both muscles was established according to findings from an Electromyography study conducted by [26]. The sensor was linked to a smartphone app, and data was sampled at a rate of 10 Hz.

The sensor operated based on the modified Beer-Lambert law to determine O₂Hb, HHb, SmO₂ as ([O₂Hb]/[tHb] *100), and Hb Diff (O₂Hb - HHb) [9, 27]. The tHb was calculated from the given values as (O₂Hb + HHb) [5]. These NIRS-derived variables (O₂Hb, HHb, Hb Diff, and tHb), expressed in micromolar (µM) units, represent indirect estimations of relative hemoglobin concentration changes derived from device-specific algorithms based on the modified Beer–Lambert law applied to NIRS. The resulting dataset for each parameter (SmO₂, O₂Hb, HHb, Hb Diff, and tHb) was then averaged across repetitions for each respective speed. This averaging approach was selected to enable between-condition comparisons; however, it is acknowledged that averaging may mask transient within-bout fluctuations in NIRS-parameters, which are dynamic variables.

### Statistical analysis

All statistical analyses were performed using SPSS (version 28.0; IBM Corp., Armonk, NY, USA). The data’s normality was assessed using visual inspection of histograms and probability plots, as well as analytical methods, specifically, the Shapiro-Wilk test. Descriptive statistics were reported for categorical variables. A two-way repeated-measures ANOVA test was used to compare the three speeds regarding oxygenation and tHb levels. It also compared CON and ECC in terms of oxygenation and tHb levels between athletes and sedentary individuals. Pairwise comparisons were performed if any significant differences were obtained. The Greenhouse –Giesser correction was applied when sphericity was violated. Effect sizes were reported as partial eta squared (ηp²) as provided by SPSS for repeated-measures ANOVA, and interpreted according to according to commonly used thresholds (small ≥ 0.01, moderate ≥ 0.06, large ≥ 0.14). The data are shown as the mean ± standard deviation. The significance level was established at α < 0.05.

## Results

### Demographic characteristics

The study found no difference in age and BMI between the athletes and healthy sedentary individuals (*p* > 0.05, Table 1).

**Table 1:** Demographic characteristics

		Volleyball Athletes (*n* = 17)	Healthy Sedentary (*n* = 14)	*p*
		(mean±SD)	(mean±SD)	
Age (years)		17.53 ± 1.50	18.21 ± 1.57	0.227
BMI (kg/m^2^)		22.9 ± 1.7	23.2 ± 4.0	0.825
		n (%)	n (%)	
Gender	*Male*	17 (54.8%)	14 (45.2%)	
	*Female*	0	0	
Lower Extremity Dominance	*Right*	16 (94.1%)	10 (71.4%)	
	*Left*	1 (5.9%)	4 (28.6%)	

*BMI* Body mass index, *p* < 0.05, *SD* Standard deviation

### Effect of isokinetic speeds during CON contractions of VL muscle

There was no Speed x Group interaction for the SmO₂, Hb Diff, tHb, and O₂Hb during CON contraction at 60, 180, and 300 °/s speed (*p* > 0.05, Table 2). Although there was no Speed x Group interaction, there was a statistical difference between the groups in terms of tHb, O₂Hb, and HHb (*p* < 0.05, Table 2). These total blood flow measures of athletes were higher than those of sedentary individuals.

**Table 2:** Effect of isokinetic speeds during CON contractions of VL Muscle in athletes and healthy sedentary individuals

		60°/s	180°/s	300°/s	Group* Speed	Group	Speed
		(mean±SD)	(mean±SD)	(mean±SD)			
**SmO₂** **(%)**	*Athletes*	63.15±11.83	62.37±11.55	63.53±11.09	F = 0.960 *p* = 0.395 ηp²=0.064	F = 0.203 *p* = 0.656 ηp²=0.007	F = 4.427 ***p*** **= 0.021** ^*^ ηp²=0.240
	*Sedentary*	63.69±8.03	63.30±7.75	66.69±7.40			
**Hb Diff** **(µM)**	*Athletes*	23.91±6.52	24.73±5.42	26.98±4.52	F = 0.973 *p* = 0.390 ηp²=0.065	F = 2.786 *p* = 0.088 ηp²0.197	F = 17.592 ***p*** **= < 0.001** ^*^ ηp²=0.557
	*Sedentary*	20.37±4.02	22.23±4.14	24.45±4.46			
**tHb** **(µM)**	*Athletes*	138.30±17.05	139.09±18.01	141.70±20.13	F = 0.949 *p* = 0.393 ηp²=0.032	F = 9.170 ***p*** **= 0.005** ^*^ ηp²=0.240	F = 9.809 ***p*** **= < 0.001** ^*^ ηp²=0.253
	*Sedentary*	116.33±22.28	117.58±22.53	118.49±23.15			
**O₂Hb** **(µM)**	*Athletes*	81.30±8.42	81.78±8.32	84.24±10.05	F = 0.585 *p* = 0.561 ηp²=0.020	F = 10.776 ***p*** **= 0.003** ^*^ ηp²=0.271	F = 16.879 ***p*** **= < 0.001** ^*^ ηp²=0.368
	*Sedentary*	68.33±12.24	69.87±12.58	71.41±12.90			
**HHb** **(µM)**	*Athletes*	57.00±10.03	57.31±10.38	57.46±10.57	F = 1.636 *p* = 0.214 ηp²=0.104	F = 6.757 ***p*** **= 0.015** ^*^ ηp²=0.189	F = 0.424 *p* = 0.659 ηp²=0.029
	*Sedentary*	48.00±10.32	47.70±10.19	47.07±10.55			

*SD* Standard Deviation, *SmO₂* Muscle oxygen saturation, *Hb Diff* Hemoglobin difference, *tHb* Total hemoglobin, *O₂Hb* Oxygenated hemoglobin, *HHb* Deoxygenated hemoglobin, *%* Percentage, *µM* Micromolar, *ηp²* Effect size

Bold values marked with * indicate statistically significant effects (*p* < 0.05)

The main effect of speed was statistically significant (*p* < 0.05, Table 2). Significant differences between speeds were observed in terms of SmO₂, Hb Diff, tHb, and O₂Hb. However, no statistically significant difference was found among the speeds in terms of HHB. The post hoc test results showed that SmO₂ during 300°/s was statistically higher than 180°/s (*p* = 0.023). No differences were observed between the other speed pairs (60 vs. 180 and 60 vs. 300). The Hb Diff levels at a speed of 60°/s exhibited a notable decrease in comparison to both 180°/s (*p* = 0.003) and 300°/s (*p* < 0.001), respectively. Also, Hb Diff at 180°/s was found to be lower than at speed 300°/s (*p* < 0.001). The tHb at 300°/s resulted in significantly higher levels compared to 60°/s (*p* < 0.001) and 180°/s (*p* = 0.007). O₂Hb levels at 300°/s exhibited a notably higher significance when compared with both 60°/s (*p* < 0.001) and 180°/s (*p* < 0.001). Conversely, there was no statistical difference in O₂Hb between 60°/s and 180°/s (*p* = 0.289).

**Fig. 1:**
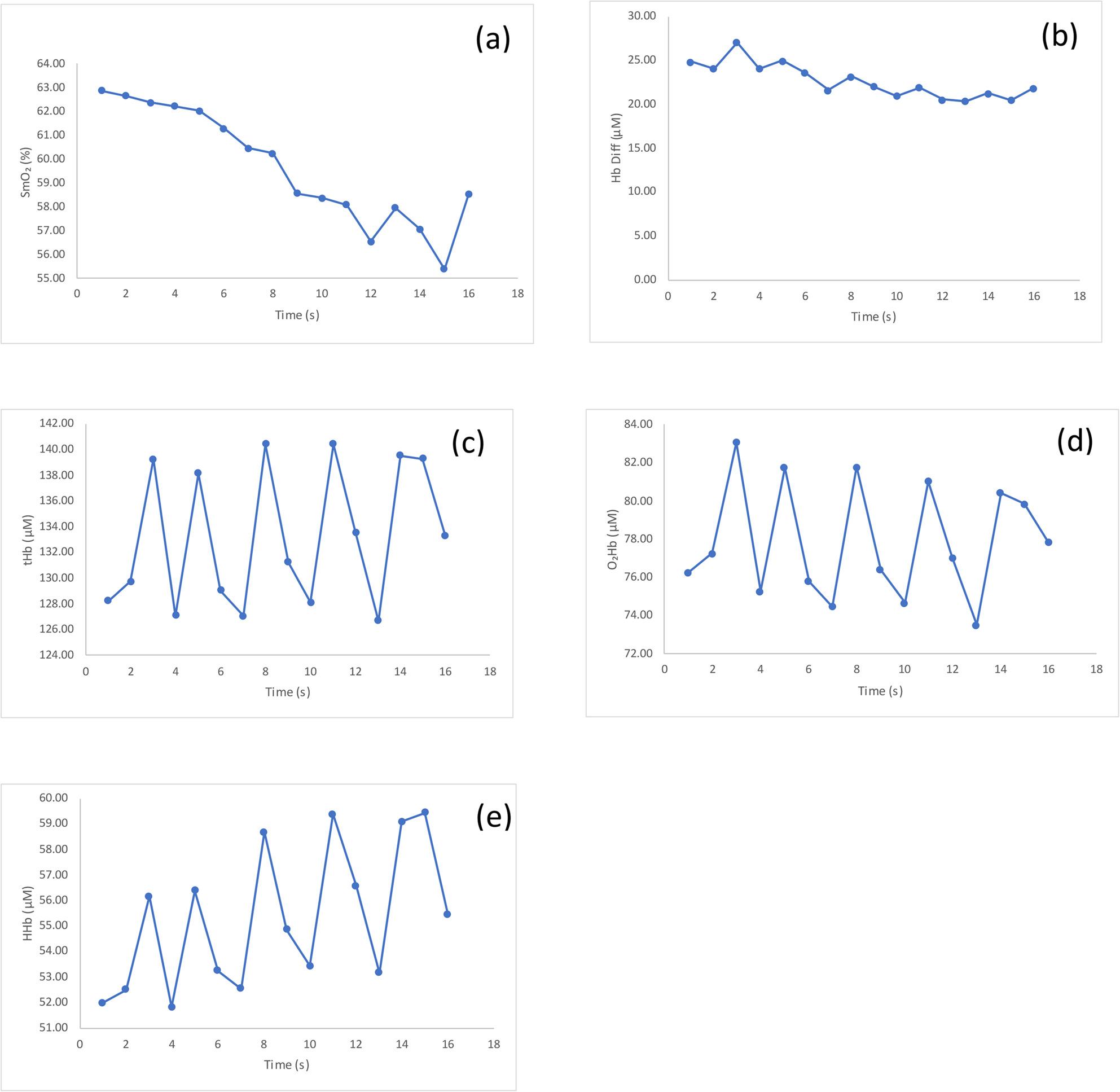
Representative profiles of NIRS-derived variables recorded during CON isokinetic contractions at 60°/s in the VL muscle of one participant: (**a**) SmO₂, (**b**) Hb Diff, (**c**) tHb, (**d**) O₂Hb, and (**e**) HHb

Figure 1 presents a representative time-series of NIRS-derived variables recorded during CON isokinetic contractions of the VL at 60°/s.

### Effect of isokinetic speeds during CON contractions of BF muscle

There was no Speed x Group interaction for the SmO₂, Hb Diff, tHb, O₂Hb, and HHb. At the same time, the group’s main effect was not statistically significant in terms of SmO₂, Hb Diff, tHb, O₂Hb, and HHb (*p* > 0.05, Table 3).

**Table 3:** Effect of isokinetic speeds during CON contractions of BF Muscle in athletes and healthy sedentary individuals

		60°/s	180°/s	300°/s	Group * Speed	Group	Speed
		(mean±SD)	(mean±SD)	(mean±SD)			
**SmO₂ (%)**	*Athletes*	67.16±9.47	69.24±10.05	68.38±10.82	F = 0.594 *p* = 0.541 ηp²=0.021	F = 0.326 *p* = 0.572 ηp²=0.012	F = 4.215 ***p*** **= 0.02** ^*^ ηp²=0.131
	*Sedentary*	69.06±5.79	70.26±6.11	70.73±5.61			
**Hb Diff (µM)**	*Athletes*	22.26±5.32	23.47±4.06	25.02±4.94	F = 0.502 *p* = 0.608 ηp²=0.018	F = 0.608 *p* = 0.442 ηp²=0.021	F = 7.591 ***p*** **= 0.001** ^*^ ηp²=0.213
	*Sedentary*	24.31±6.92	25.23±6.66	25.97±6.99			
**tHb** **(µM)**	*Athletes*	125.70±19.84	125.94±20.20	131.30±22.44	F = 1.423 *p* = 0.249 ηp²=0.048	F = 2.506 *p* = 0.125 ηp²=0.082	F = 1.182 *p* = 0.314 ηp²=0.041
	*Sedentary*	116.58±22.18	115.21±21.73	115.67±21.21			
**O** **₂** **Hb (μM)**	*Athletes*	73.95±10.51	74.64±10.34	77.85±11.16	F = 1.453 *p* = 0.243 ηp²=0.049	F = 1.342 *p* = 0.256 ηp²=0.046	F = 2.402 *p* = 0.100 ηp²=0.079
	*Sedentary*	70.44±14.21	70.17±13.89	70.80±13.82			
**HHb (µM)**	*Athletes*	51.74±10.02	51.30±10.26	53.45±11.66	F = 1.194 *p* = 0.305 ηp²=0.041	F = 0.533 *p* = 0.558 ηp²=0.019	F = 4.155 *p* = 0.061 ηp²=0.129
	*Sedentary*	46.14±8.24	45.03±8.05	44.87±7.62			

*SD* Standard Deviation, *SmO₂* Muscle oxygen saturation, *Hb Diff* Hemoglobin difference, *tHb* Total hemoglobin, *O₂Hb* Oxygenated hemoglobin, *HHb* Deoxygenated hemoglobin, *%* Percentage, *µM* Micromolar, *ηp²* Effect size

Bold values marked with * indicate statistically significant effects (*p* < 0.05)

The main effect of speed was significant in terms of SmO₂ and Hb Diff (*p* < 0.05, Table 3). Hb Diff was significantly greater during 300°/s compared to 60°/s (*p* = 0.008). Nevertheless, no statistically significant differences were found between 60 and 180°/s (*p* = 0.102) and between 180 and 300°/s (*p* = 0.131). The overall speed effect on SmO₂ was statistically significant; however, subsequent pairwise comparisons (post-hoc tests) did not reveal any specific differences between conditions.

### Effect of isokinetic speeds during CON/ECC contractions of VL muscle

No Contraction x Group interaction existed for SmO₂, Hb Diff, tHb, O₂Hb, and HHb during CON and ECC 60°/s (*p* > 0.05, Table 4). Notably, there was a difference between the groups in terms of the Hb Diff, tHb, O₂Hb, and HHb (*p* < 0.05, Table 4). Total blood flow measures of athletes were higher than those of sedentary individuals.

**Table 4:** Effect of isokinetic speeds during CON/ECC contractions of VL muscle in athletes and healthy sedentary individuals

		60°/s	60°/s	Group * Contraction	Group	Contraction
		CON	ECC			
		(mean±SD)	(mean±SD)			
**SmO₂** **(%)**	*Athletes*	63.15±11.83	63.60±9.19	F = 1,317 *p* = 0.261 ηp²=0.043	F = 0.677 *p* = 0.417 ηp²=0.023	F = 2.037 *p* = 0.164 ηp²=0.066
	*Sedentary*	63.69±8.03	67.81±5.61			
**Hb Diff** **(µM)**	*Athletes*	23.91±6.52	29.97±5.06	F = 0.212 *p* = 0.649 ηp²=0.007	F = 4.536 ***p*** **= 0.042** ^*^ ηp²=0.135	F = 97.420 ***p*** **= < 0.001** ^*^ ηp²=0.771
	*Sedentary*	20.37±4.02	25.89±4.58			
**tHb** **(µM)**	*Athletes*	138.30±17.05	143.43±18.21	F = 0.103 *p* = 0.750 ηp²=0.004	F = 9.520 ***p*** **= 0.004** ^*^ ηp²=0.247	F = 27.307 ***p*** **= < 0.001** ^*^ ηp²=0.485
	*Sedentary*	116.33±22.28	120.87±23.45			
**O₂Hb** **(µM)**	*Athletes*	81.30±8.42	86.65±9.13	F = 0.636 *p* = 0.537 ηp²=0.043	F = 11.838 ***p*** **= 0.002** ^*^ ηp²=0.290	F = 19.136 ***p*** **= < 0.001** ^*^ ηp²=0.577
	*Sedentary*	68.33±12.24	73.36±13.42			
**HHb** **(µM)**	*Athletes*	57.00±10.03	56.78±9.77	F = 0.201 *p* = 0.658 ηp²=0.007	F = 6.348 ***p*** = 0.018* ηp²=0.180	F = 1.453 *p* = 0.238 ηp²=0.048
	*Sedentary*	48.00±10.32	47.51±10.25			

*SD* Standard Deviation, *SmO₂* Muscle oxygen saturation, *Hb Diff* Hemoglobin difference, *tHb* Total hemoglobin, *O₂Hb* Oxygenated hemoglobin, *HHb* Deoxygenated hemoglobin, *%* Percentage, *µM* Micromolar, *ηp²* Effect size

Bold values marked with * indicate statistically significant effects (*p* < 0.05)

The contraction’s main effect was statistically significant (*p* < 0.05, Table 4). Significant differences were observed regarding speeds between Hb Diff, tHb, and O₂Hb. However, no statistical difference was observed among the contractions regarding HHB. According to the pairwise comparison, Hb Diff, tHb, and O₂Hb were higher in CON at 60°/s than in ECC at 60°/s.

### Effect of isokinetic speeds during CON/ECC testing of BF muscle

During CON and ECC 60°/s, Contraction x Group interaction was only for the Hb Diff. The Hb Difference was higher for athletes (< 0.001) during CON and ECC. Although there was Contraction x Group interaction for the Hb Diff, the group’s main effect was not statistically significant (*p* > 0.05, Table 5).

**Table 5:** Effect of isokinetic speeds during CON/ECC contractions of BF muscle in athletes and healthy sedentary individuals

		60°/s	60°/s	Group * Contraction	Group	Contraction
		CON	ECC			
		(mean±SD)	(mean±SD)			
**SmO₂** **(%)**	*Athletes*	67.16±9.47	69.57±10.87	F = 0.581 *p* = 0.452 ηp²=0.020	F = 0.053 *p* = 0.819 ηp²=0.002	F = 0.471 *p* = 0.498 ηp²=0.017
	*Sedentary*	69.06±5.79	68.93±6.18			
**Hb Diff** **(µM)**	*Athletes*	22.26±5.32	30.74±4.32	F = 6.418 ***p*** **= 0.017** ^*^ ηp²=0.186	F = 0.015 *p* = 0.903 ηp²=0.001	F = 48.066 ***p*** **= < 0.001** ^*^ ηp²=0.632
	*Sedentary*	24.31±6.92	28.25±5.69			
**tHb** **(µM)**	*Athletes*	125.70±19.84	129.84±19.09	F = 1.077 *p* = 0.355 ηp²=0.074	F = 1.587 *p* = 0.218 ηp²=0.054	F = 0.904 *p* = 0.417 ηp²=0.063
	*Sedentary*	116.58±22.18	120.39±20.98			
**O₂Hb** **(µM)**	*Athletes*	73.95±10.51	80.28±11.00	F = 1.602 *p* = 0.216 ηp²=0.054	F = 1.202 *p* = 0.282 ηp²=0.041	F = 28.029 ***p*** **= < 0.001** ^*^ ηp²=0.500
	*Sedentary*	70.44±14.21	74.32±12.75			
**HHb** **(µM)**	*Athletes*	51.74±10.02	49.56±8.39	F = 1.905 *p* = 0.178 ηp²=0.064	F = 2.033 *p* = 0.165 ηp²=0.068	F = 2.172 *p* = 0.152 ηp²=0.072
	*Sedentary*	46.14±8.24	46.07±8.59			

*SD* Standard Deviation, *SmO₂* Muscle oxygen saturation, *Hb Diff* Hemoglobin difference, *tHb* Total hemoglobin, *O₂Hb* Oxygenated hemoglobin, *HHb* Deoxygenated hemoglobin, *%* Percentage, *µM* Micromolar, *ηp²* Effect size

Bold values marked with * indicate statistically significant effects (*p* < 0.05)

The main effect of speed was statistically significant (*p* < 0.05, Table 5). Significant differences were observed in the Hb Diff and O₂Hb between contractions. Nonetheless, no statistically notable differences were detected among the contractions concerning SmO₂, tHb, and HHb. According to the post hoc test results, Hb Diff and O₂Hb in the 60°/s ECC were statistically higher than in the speed CON 60°/s (*p* < 0.001).

## Discussion

Our findings suggest that contraction velocity has a significant influence on muscle oxygenation and local blood volume in the quadriceps (VL), consistent with established exercise physiology. At 300°/s, the VL showed higher SmO₂ and tHb than at slower speeds. In slow-velocity muscle actions, both slow-twitch (type I) and fast-twitch (type II) muscle fibers are likely recruited, resulting in greater muscle activity, increased intramuscular pressure, and higher energy demand, which may reduce tissue oxygenation. In contrast, high-speed contractions engage fewer fibers (mainly type I), resulting in relatively higher SmO₂ [28].

Consistent with these observations, a previous study that explored the impact of angular velocity on oxygen levels within the VL and rectus femoris muscles [29] reported that the level of SmO₂ rose proportionally with angular velocities of 60°, 120°, and 240°/s. While that study also reported an inverse relationship between tHb and velocity, our study found an increase in tHb with higher velocity. We believe that these differences may be attributed to the varying velocities used in their study compared to those used in our study, as well as population differences.

Elevated tHb and O₂Hb at high contraction speed may indicate greater local blood volume. One hypothesis is that the rapid rhythmic compression and relaxation of blood vessels by muscle contractions act as a “muscle pump,” which could enhance venous return and facilitating the circulation of blood back to the heart [30]. An alternative explanation is that during physical activity, the sympathetic nervous system may increase cardiac output and redistributes blood flow by releasing norepinephrine, which binds to α-adrenergic receptors on a smooth vascular muscle, leading to vasoconstriction in less active tissues [31].

In the present study, HHb values did not show significant changes across speeds despite changes in SmO₂ and tHb. Our findings corroborate a pervious study [32], which observed that HHb levels remained constant at moderate work rates despite continual increases in tHb, O₂Hb. Although HHb is commonly considered as a marker of muscle oxygen extraction, its responsiveness during dynamic isokinetic exercise may be constrained by concurrent changes in blood volume, motion-related signal artefacts, and the averaging of rapidly changing signals across repetitions. Under such conditions, HHb may remain relatively stable despite alterations in oxygen delivery and utilization. Significantly, this does not diminish the physiological relevance of HHb; instead, it suggests that HHb alone may be insufficient to characterize muscle oxygen extraction in dynamic tasks. We emphasize that any interpretation of metabolic cost from these NIRS signals is tentative, and the interpretation of muscle oxygenation is strengthened when HHb is evaluated in conjunction with additional NIRS-derived indices.

Contractile mode also influenced the results [29]. CON work generally demands higher oxygen consumption and recruits more motor units than ECC exercises [33]. At 60°/s, we found higher tHb, O₂Hb, and Hb Diff in CON compared to ECC in the VL, with no significant differences in SmO₂ and HHb, consistent with prior observations [34]. The heightened metabolic demands during CON likely necessitate increased blood flow [34]. This may involve pronounced vasodilation and blood vessel recruitment to meet the demands of active muscle fibers [35] reflected in our higher O₂Hb and tHb findings. Interestingly, in the BF we observed the opposite pattern: at 60°/s, Hb Diff and O₂Hb were higher during ECC than CON. This novel finding possibly due to the anatomical and distinct muscle recruitment patterns in hamstrings.

Athletes showed higher tHb and O₂Hb responses in the VL than sedentary individuals. This may be due to training adaptations, such as increased capillary density and enhanced oxidative enzyme activity [36]. This can also be possibly linked to reduced intima-media thickness and improved arterial compliance observed in athletes, as reported in a previous study. This suggests that the training status may modulate NIRS signals, but the fundamental velocity- and mode-dependent patterns were similar in both groups.

In the BF, group differences were minimal, with only Hb Diff being specifically higher, hinting at better oxygen extraction. While the absence of significant differences may seem unexpected, it does not negate the existence of baseline differences between athletes and sedentary individuals. These similar oxygenation and blood flow responses across speeds and between groups suggest that the BF muscle has a relatively stable oxygen demand. To impose a greater metabolic challenge on the hamstrings, practitioners might emphasize eccentric or heavier-load exercises, which may increase strain and oxygen demand in these muscles.

In summary, the primary contribution of this study is physiological. We provide new insight into how isokinetic contraction velocity and mode alter local muscle oxygenation. The data support classic mechanisms (greater O₂ demand in CON vs. ECC) and extend them by quantifying SmO₂ and tHb changes across multiple speeds and muscles. Methodologically, we also present one of the first NIRS characterizations of the biceps femoris during dynamic exercise. We recommend using combined metrics (SmO₂ or HbDiff along with O₂Hb and HHb) to obtain a more complete assessment of muscle oxygenation (recognizing that these are still indirect indices).

In addition, inter-individual variability was minimized through strict inclusion criteria and a within-subject experimental design, with each participant completing all contraction velocities and modes. Experimental conditions, including NIRS sensor placement and rest intervals, were standardized. Athlete and sedentary groups were comparable in age and sex and were assessed using identical protocols, ensuring that observed differences primarily reflect physiological or training-related factors rather than methodological variability.

Several limitations of our study should be acknowledged. First, there is a lack of validation studies for the Train.Red FYER sensor in sport sciences, as it is a relatively new device. Therefore, the present findings should be interpreted as exploratory within-device observations.

Second, the sample size was small and consisted solely of male participants, and analyses were restricted to the dominant leg, which may limit the generalizability of the findings. Future studies should include larger and more diverse samples (including female participants) and incorporate power calculations to ensure the robustness of these results.

Third, despite randomization of contraction order and standardized rest intervals, the absence of direct fatigue assessment limits our ability to entirely exclude fatigue-related influences on NIRS-derived oxygenation responses.

Fourth, NIRS methodology has inherent limitations. NIRS signals may be affected by motion artifacts during high-speed contractions and by changes in local blood volume influencing O₂Hb and HHb; thus, even with the use of Hb Diff, NIRS variables should be considered indirect indicators of muscle oxygenation rather than a direct measure of oxygen consumption.

Fifth, our analysis used averaged SmO₂, tHb, and NIRS-derived parameter values for each contraction set. However, SmO₂ and HHb are dynamic signals that change during the contraction–recovery cycle. Averaging may mask early or transient changes, such as an initial SmO₂ drop at the onset of contraction or rapid reoxygenation afterward. Future analyses should examine the full-time course of these signals during each contraction, to capture the kinetics of oxygenation.

Finally, we did not directly measure muscle blood flow or metabolic cost. NIRS signals are indirect; incorporating direct measures (such as Doppler ultrasound for blood flow, EMG for activation) would help link tHb and SmO₂ changes to actual perfusion and muscle activity. Similarly, quantifying oxygen consumption (VO₂) or metabolites (e.g., lactate) would clarify the energetic implications of different speeds. Studying muscle-specific factors (such as fiber-type distribution or capillary density via imaging or biopsy) could explain the distinct responses in VL vs. BF. In addition, applying similar protocols to training or rehabilitation interventions (especially hamstring-focused exercises) would help test the hypotheses generated here.

## Conclusion

In summary, the results demonstrate that isokinetic speed and contraction mode significantly influence muscle oxygenation and blood volume in a muscle-dependent way. High-speed contractions enhanced quadriceps oxygen saturation and perfusion, while hamstring oxygenation remained comparatively stable. CON versus ECC actions also elicited distinct oxygenation profiles in each muscle. We did not assess fatigue or recovery, so we do not infer effects beyond the observed physiological responses. Overall, mapping these muscle-specific oxygenation dynamics provides a basis for designing exercise or rehabilitation protocols that consider how speed and contraction type affect local oxygen delivery and use.

## Data Availability

Data is available from the corresponding author upon request.

## Abbreviations

ATT: Adipose tissue thickness
BF: Biceps femoris
BMI: Body mass index
CON: Concentric
ECC: Eccentric
Hb Diff: Hemoglobin difference
HHb: Deoxygenated hemoglobin
IPAQ: International Physical Activity Questionnaire
ISAK: International society for the advancement of kinanthropometry
NIRS: Near-infrared spectroscopy
O₂Hb: Oxygenated hemoglobin
SmO₂: Muscle oxygen saturation
tHb: Total hemoglobin
VL: Vastus lateralis
